# Integrative Analysis Prioritizes CRIP2 as a Candidate Associated with Myocardial Copper-Handling Responses After Myocardial Infarction

**DOI:** 10.3390/cimb48080767

**Published:** 2026-07-28

**Authors:** Zhengqi Qiu, Xingya Lei, Xueqin Zhang

**Affiliations:** 1 Department of Applied Psychology, School of Health Management, Guangzhou Medical University, Guangzhou 510182, China; caspiansev@163.com (Z.Q.); xingyal3650@163.com (X.L.); 2 Guangdong Engineering Technology Research Center for Translational Medicine of Mental Disorders, Guangzhou Medical University, Guangzhou 510260, China

**Keywords:** myocardial infarction, mendelian randomization, single-cell RNA sequencing, CRIP2, copper homeostasis

## Abstract

Post-myocardial infarction (MI) remodeling is accompanied by metabolic stress, but the myocardial genes associated with copper handling are poorly defined. We sought to prioritize a tissue-derived candidate rather than establish a copper-dependent mechanism. Regional MI transcriptomes and an in-house left anterior descending coronary artery ligation mouse RNA-sequencing cohort were integrated with protein quantitative trait locus-based Mendelian randomization (MR). Follow-up comprised local and external tissue validation, cardiac single-cell RNA sequencing, Genotype-Tissue Expression co-expression, computational perturbation, and CRIP2 knockdown or overexpression in H9c2 cells exposed to hypoxia/reoxygenation (H/R). CRIP2 showed a nominal protective-direction MR association (odds ratio 0.831, 95% confidence interval 0.735–0.939; *p* = 0.0031), but did not pass the Bonferroni threshold. *Crip2* was lower in the local MI model (*p* = 0.0168; *n* = 5 per group) and in an independent dataset. A prespecified lipoylated-tricarboxylic-acid module was negatively enriched after MI (normalized enrichment score −1.63; false discovery rate 0.012), whereas the broader copper-homeostasis set was not significant. Single-cell data localized *Crip2* mainly to cardiomyocytes, but were not adequately replicated for condition-level inference. Under H/R, *Atp7a* was the only copper-handling transcript whose knockdown-by-oxygen interaction remained significant after adjustment (q = 0.0405). CRIP2 overexpression was associated with higher Cell Counting Kit-8 metabolic activity during H/R (interaction *p* = 0.00551), whereas the knockdown interaction was not significant. Copper abundance, mitochondrial function, and cuproptosis markers were not measured. The data prioritize CRIP2 for mechanistic study, but do not show that it regulates copper flux or post-MI remodeling.

## 1. Introduction

Ischemic heart disease remained the leading cause of age-standardized disability-adjusted life years in 2022 [1]. Survival after acute myocardial infarction (MI) has improved, yet long-term studies show persistent cardiovascular and noncardiovascular risk [2,3]. In a cohort of 433,361 people with MI, 38% died within nine years, nearly one-third developed heart or renal failure, and 7% had recurrent MI [4]. These outcomes motivate work on chronic repair, in addition to acute care and secondary prevention [5,6,7]. Cardiomyocyte loss, inflammation, and fibroblast-driven matrix remodeling contribute to adverse ventricular remodeling [8,9,10]. Copper homeostasis is one candidate metabolic axis [11]: copper supports mitochondrial respiration and antioxidant defense through enzymes including cytochrome c oxidase and SOD1 [12,13], whereas excess copper can disrupt electron transport, increase reactive oxygen species, and trigger cuproptosis through lipoylated-protein aggregation [14].

Experimental studies link copper excess to mitochondrial injury and cardiomyocyte death [15]. SIRT3-dependent signaling [16] and copper-handling proteins such as COX17 and ATP7A/B [17] have also been implicated in cardiac copper responses. These observations justify examining copper-related pathways in the infarcted heart, while leaving open which myocardial genes are relevant in vivo.

Most cardiac copper studies emphasize systemic measures or single experimental models, which cannot resolve region- and cell-type-specific responses within the injured myocardium [18]. CRIP2 was not selected a priori; it emerged from our tissue-anchored screen. The protein contains LIM and cysteine-rich domains and has been implicated in vascular development [19]. It has also been identified as a nuclear copper-binding partner of ATOX1, with copper-dependent effects on reactive oxygen species and autophagy [20]. This literature made CRIP2 a plausible follow-up candidate, but did not establish a role in MI.

The objective of this study was to identify a reproducible myocardial candidate, determine its cardiac cell-type distribution, and ask whether genetic, computational, and cell-based data supported further mechanistic testing. Protein quantitative trait locus MR was used for prioritization [21]; single-cell and in silico analyses were treated as complementary, hypothesis-generating evidence [22,23,24], not as proof of causality.

## 2. Results

### 2.1. Integrated MI Transcriptomics and pQTL-MR Nominally Prioritize CRIP2

**Figure 1:**
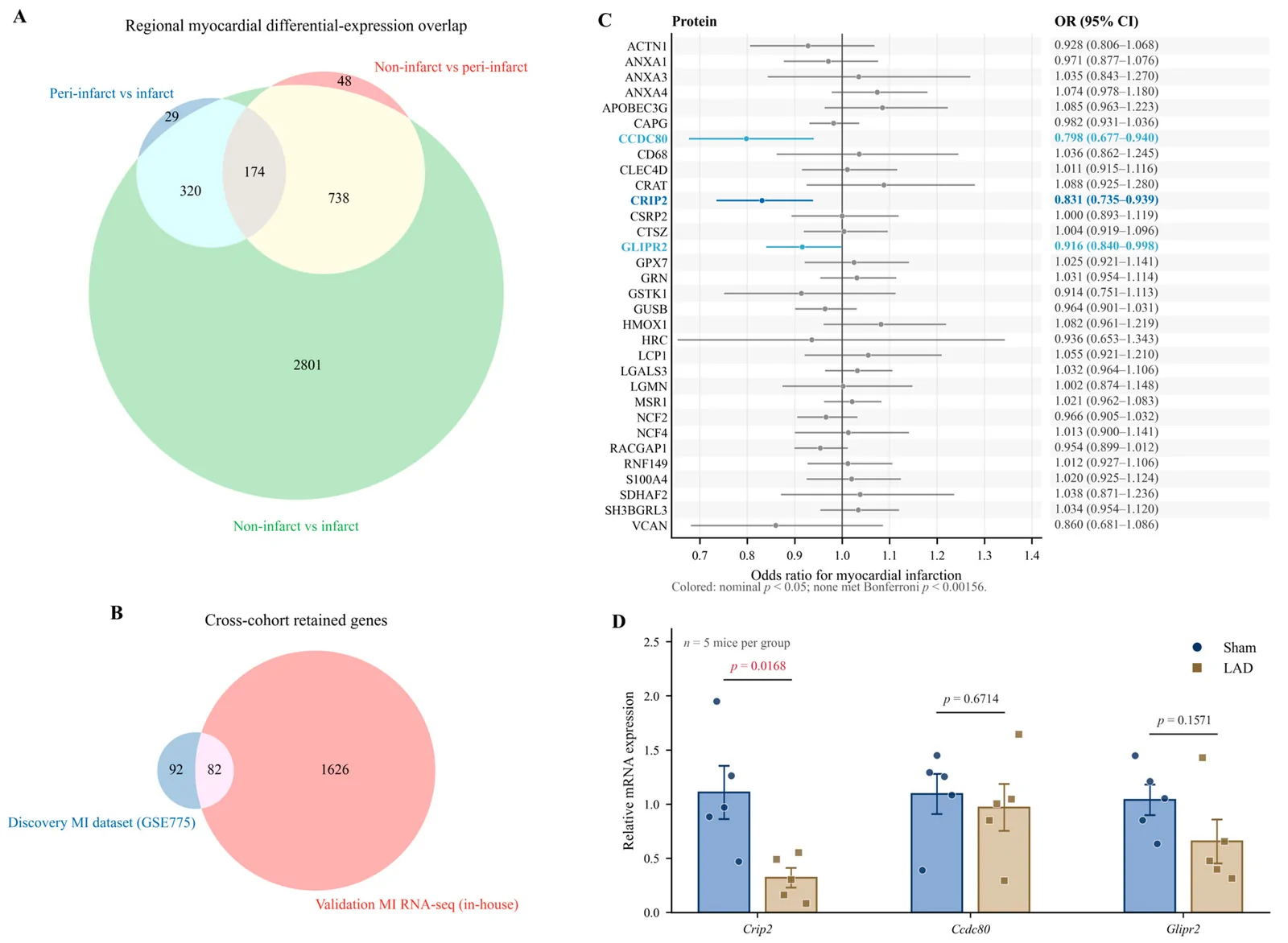
Multi-cohort transcriptomic integration and proteomic Mendelian randomization nominally prioritize CRIP2 as a candidate downregulated in myocardial infarction. (**A**) Intersection of differentially expressed genes across infarcted myocardium (IM), peri-infarct zone (PZ), and non-infarcted myocardium (NI) in GSE775, yielding 174 shared genes. (**B**) Intersection of the 174 GSE775 genes with the in-house mouse MI RNA-sequencing cohort, yielding 82 shared genes. (**C**) Two-sample MR estimates for circulating candidate protein levels and MI risk. IVW estimates were CRIP2: OR 0.831 (95% CI 0.735–0.939), *p* = 0.00306, 12 variants; CCDC80: OR 0.798 (95% CI 0.677–0.940), *p* = 0.00702, 19 variants; and GLIPR2: OR 0.916 (95% CI 0.840–0.999), *p* = 0.0461, 20 variants. These associations were nominal (*p* < 0.05), and none met the Bonferroni threshold (*p* < 0.00156). (**D**) Quantitative real-time PCR validation in the local mouse LAD-ligation MI model at four weeks after MI. Data are mean ± SEM (*n* = 5 mice per group for each gene; exact *p* values are displayed; two-tailed unpaired Student’s *t*-test). Panel colors and symbols are defined as follows: colors in (**A**,**B**) correspond to the labeled comparisons or datasets; blue estimates identify the three nominally associated candidates and gray estimates show the remaining screened proteins in (**C**); blue circles/bars denote sham and tan squares/bars denote left anterior descending (LAD) coronary artery ligation in (**D**). Abbreviations: MR, Mendelian randomization; IVW, inverse-variance weighted; OR, odds ratio.

Pairwise differential-expression analyses across infarcted myocardium (IM), the peri-infarct zone (PZ), and non-infarcted myocardium (NI) in GSE775 identified a 174-gene triple overlap (Figure 1A). Intersection with our in-house mouse LAD/MI RNA-sequencing cohort yielded 82 shared genes (Figure 1B). Among approximately 32 proteins screened by pQTL-MR, three had nominal protective-direction IVW estimates: CRIP2 (12 variants; OR 0.831, 95% CI 0.735–0.939, *p* = 0.00306), CCDC80 (19 variants; OR 0.798, 95% CI 0.677–0.940, *p* = 0.00702), and GLIPR2 (20 variants; OR 0.916, 95% CI 0.840–0.999, *p* = 0.0461; Figure 1C). None met the Bonferroni threshold of *p* < 0.00156. CRIP2 was prioritized for follow-up because all instruments were strong (minimum F = 20.96; mean F = 41.96), with no marked heterogeneity (IVW Cochran Q *p* = 0.900), directional pleiotropy (MR-Egger intercept *p* = 0.357), or MR-PRESSO global evidence of horizontal pleiotropy (*p* = 0.902). Directionally consistent sensitivity estimates were obtained by weighted median (OR 0.837, *p* = 0.0331) and MR-Egger (OR 0.751, *p* = 0.0413). The stringent three-SNP IVW model remained directionally consistent (OR 0.815, 95% CI 0.689–0.963, *p* = 0.0165), and leave-one-out estimates ranged from OR 0.814 to 0.844 (maximum *p* = 0.0140; Figure S1). In contrast, the only available cis-window candidate, rs4983411, was null (Wald-ratio OR 0.937, 95% CI 0.545–1.610, *p* = 0.814), reinforcing that the MR finding is supportive, rather than definitive, causal evidence. In the local LAD-ligation MI model, *Crip2* mRNA was reduced four weeks after MI (*p* = 0.0168; *n* = 5 per group), whereas *Ccdc80* and *Glipr2* did not reach statistical significance (Figure 1D). Independent GSE236374 analysis confirmed marked *Crip2* downregulation at MI day 7 versus sham (log2 fold change = −1.810; adjusted *p* = 4.23 × 10^−84^) [25] (Figure S2). The seven-gene cuproptosis-permissive/lipoylated-TCA core module was negatively enriched (NES = −1.633; nominal *p* = 0.00457; FDR = 0.0121), whereas the broader 14-gene copper-homeostasis set was not significant (NES = −1.295; FDR = 0.236). This pattern is compatible with coordinated transcriptional suppression and candidate prioritization; it is not evidence of cuproptotic cell death or causality.

### 2.2. Single-Cell Profiling Localizes Crip2 to Cardiomyocytes After MI

**Figure 2:**
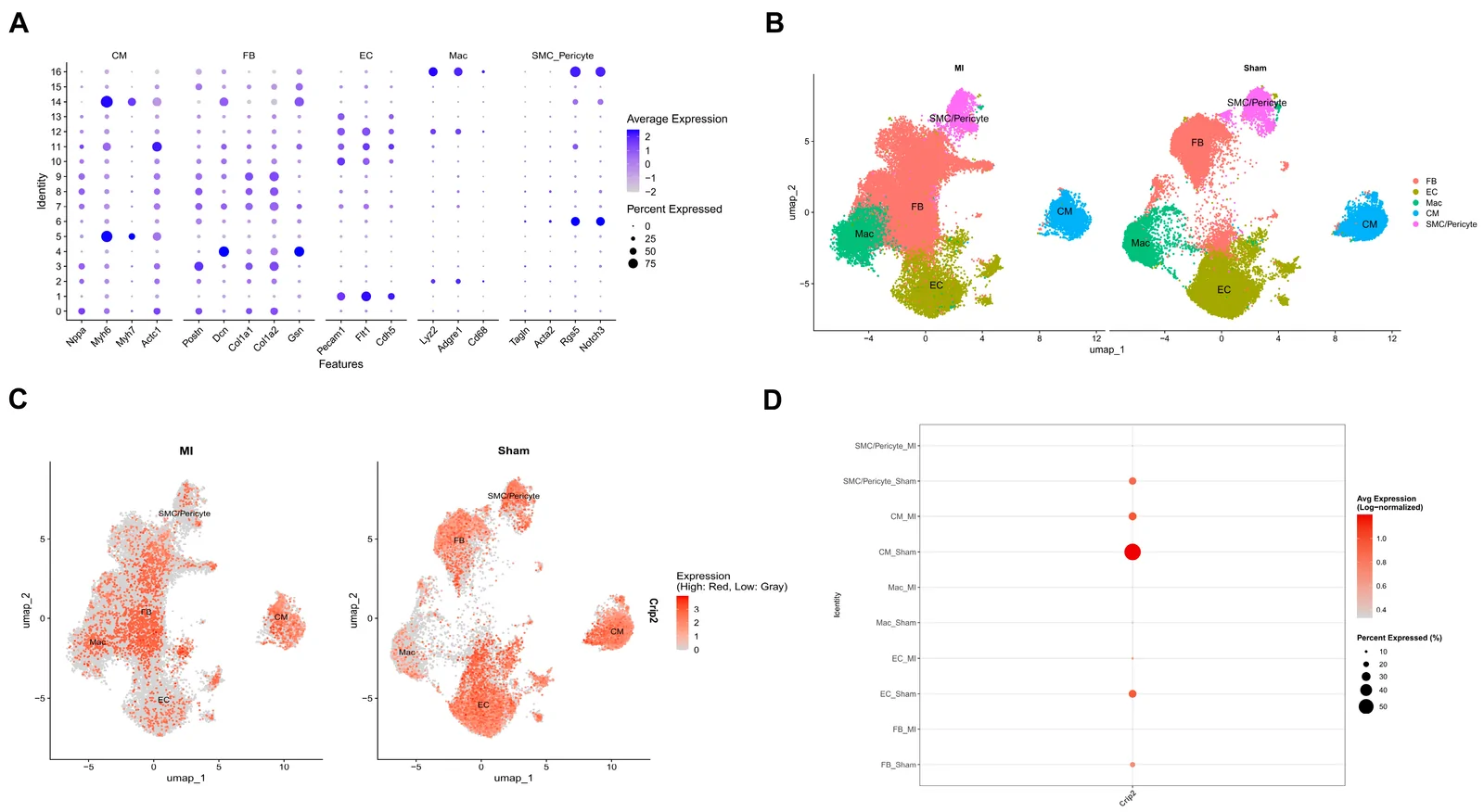
Single-cell transcriptomic profiling maps Crip2 expression across cardiac cell populations after myocardial infarction. (**A**) Dot plot of canonical marker genes used to annotate five major cardiac cell populations: cardiomyocytes (CM), fibroblasts (FB), endothelial cells (EC), macrophages (Mac), and smooth muscle cells/pericytes (SMC/Pericyte). Dot size and color indicate the percentage of expressing cells and average expression level, respectively. (**B**) UMAP visualization of cardiac single-cell transcriptomes split by condition (MI vs. Sham). (**C**) Feature plots map Crip2 expression density and show a descriptively lower signal after MI. (**D**) Dot plot comparing Crip2 expression across cell populations and conditions. Because two nominal MI raw matrices were byte-identical, the unique-heart pseudobulk sensitivity analysis included three sham and two MI hearts, and did not detect a significant condition-level Crip2 difference (exact permutation *p* = 0.40); the atlas is therefore interpreted as cell-type localization evidence, rather than replicated proof of post-MI downregulation. In (**B**), colors denote annotated cell types; in (**C**), red and gray indicate higher and lower Crip2 expression, respectively; and in (**D**), color intensity and dot size indicate mean expression and the percentage of expressing cells. UMAP, Uniform Manifold Approximation and Projection.

The originally processed GSE214611 atlas resolved five major populations using canonical markers: cardiomyocytes, fibroblasts, endothelial cells, macrophages, and smooth muscle cells/pericytes (Figure 2A,B). Crip2 expression was broad but descriptively highest in cardiomyocytes under sham conditions, with reduced expressing-cell proportions in MI across cardiomyocytes and several stromal/vascular populations (Figure 2C,D). However, raw-file integrity auditing showed that the nominal MI accessions GSM6613074_snd7_1 and GSM6613075_snd7_2 were byte-identical at the matrix, feature, and barcode levels. Unique-heart pseudobulk sensitivity analysis therefore comprised three sham and two unique MI hearts. The MI-minus-sham differences were −0.498 log2 counts per million for Crip2 (exact permutation *p* = 0.40), −1.329 for the seven-gene core-module score (*p* = 0.10), and +0.091 for the 14-gene copper-homeostasis module (*p* = 0.90). Accordingly, the single-cell results are used for descriptive cell-type localization only, and do not provide adequately replicated condition-level inference.

### 2.3. Exploratory Computational Models Yield Non-Concordant CRIP2 Perturbation Signals

**Figure 3:**
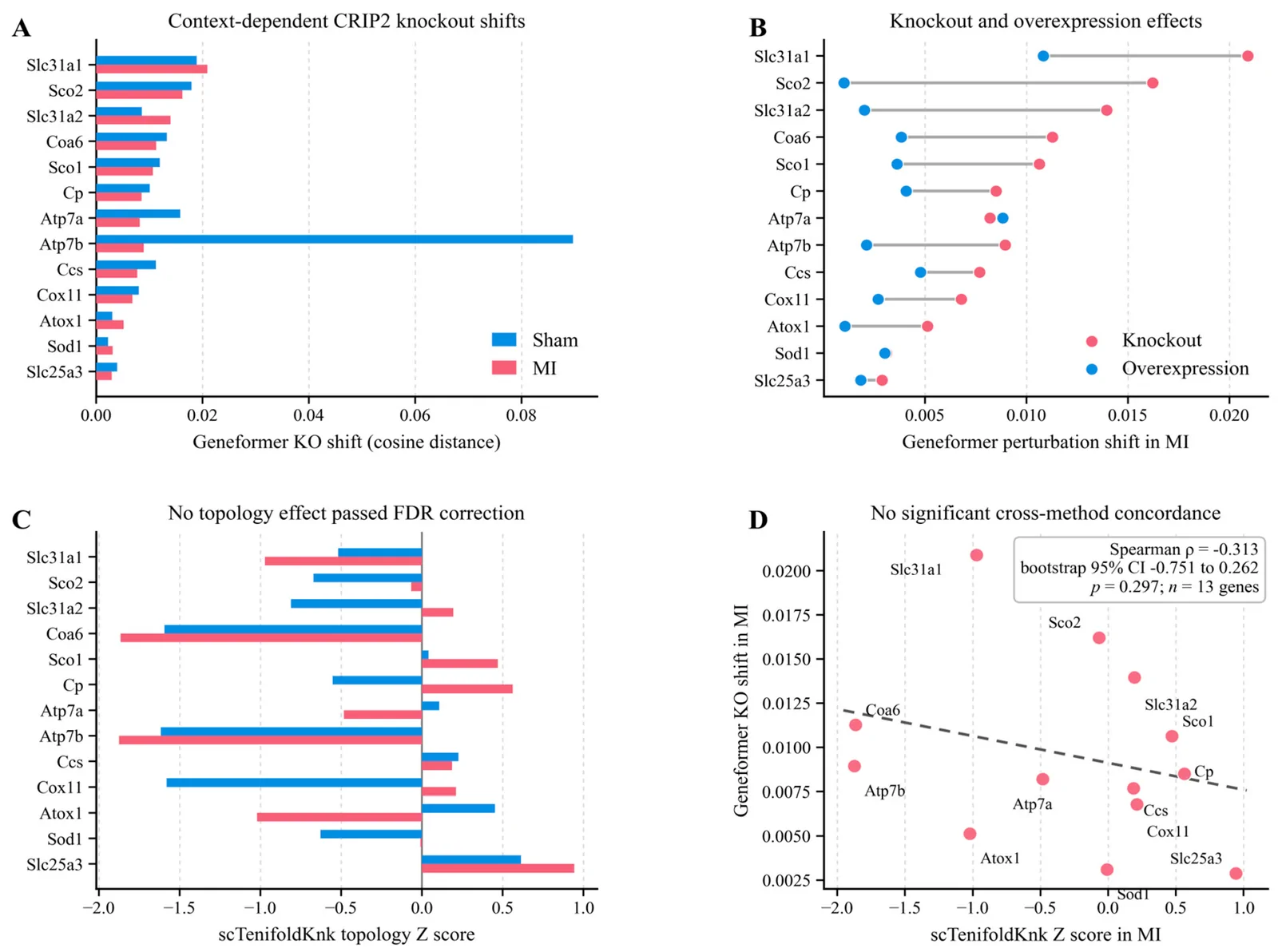
Exploratory in silico modeling of CRIP2 perturbation and downstream copper-handling network responses after myocardial infarction. (**A**) Geneformer embedding shifts (cosine distance) among copper-handling and mitochondrial respiratory genes after virtual CRIP2 perturbation in MI and sham contexts. (**B**) Comparison of virtual CRIP2 knockout (KO) and overexpression (OE) effects; KO effects were often, but not invariably, larger than OE effects. (**C**) scTenifoldKnk topology Z-scores after virtual CRIP2 knockout in MI and sham contexts; no adjusted-significant topology effects were identified. (**D**) Exploratory comparison of Geneformer KO_MI embedding shifts with scTenifoldKnk Z_MI scores across 13 genes (Spearman ρ = −0.313, 95% bootstrap CI −0.751 to 0.262, *p* = 0.297; 100,000 resamples). The non-significant association does not support cross-method concordance. Cox17 was not included because it did not pass the Geneformer top-2048-gene input threshold. Blue and red denote sham and MI in (**A**,**C**), whereas blue and red denote overexpression and knockout in (**B**). Red points in (**D**) represent individual genes, and the dashed line is the fitted trend. FDR, false discovery rate.

Virtual CRIP2 knockout and overexpression were modeled in MI and sham single-cell contexts, with downstream changes summarized across 13 copper-handling and mitochondrial respiratory genes (Figure 3A,B). Geneformer embedding shifts differed by gene and condition; knockout shifts were often, but not consistently, larger than overexpression shifts. scTenifoldKnk yielded descriptive topology Z-scores, but no gene-level effect remained significant after adjustment (Figure 3C). The Geneformer and scTenifoldKnk outputs were not significantly correlated (Spearman rho = −0.313, 95% bootstrap confidence interval −0.751 to 0.262, *p* = 0.297; *n* = 13; Figure 3D). The models therefore generated distinct exploratory signals, rather than convergent evidence of network disruption.

### 2.4. Selected Transcripts Change After CRIP2 Perturbation in H9c2 Cells

**Figure 4:**
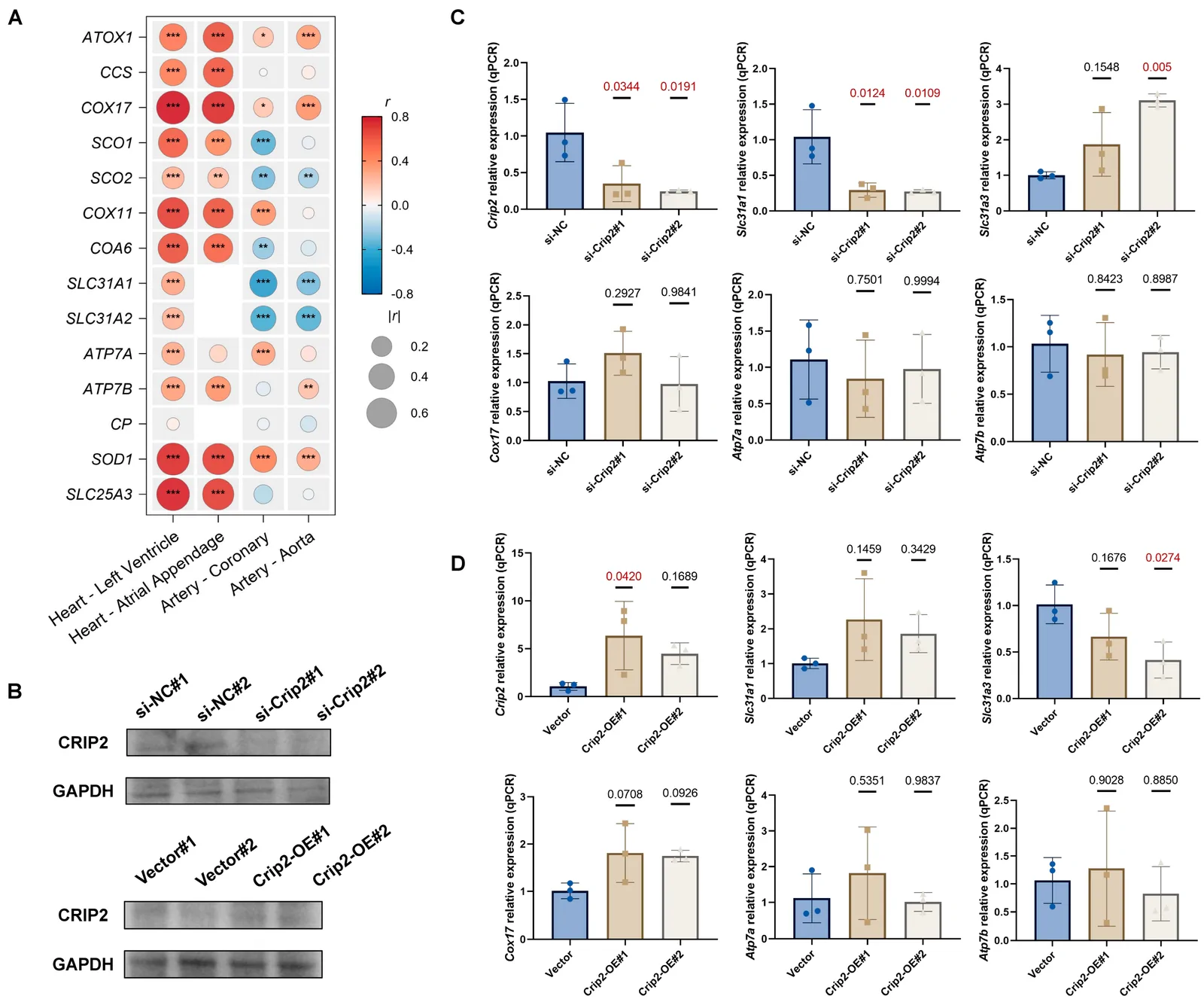
CRIP2 perturbation alters selected copper-handling transcripts in H9c2 cardiomyoblasts. (**A**) Bubble heatmap of GTEx co-expression correlations between CRIP2 and 14 copper-handling genes across human cardiovascular tissues; circle size represents |r|. * *p* < 0.05, ** *p* < 0.01, *** *p* < 0.001. (**B**) Western blots confirming CRIP2 knockdown and overexpression; GAPDH was the loading control. (**C**,**D**) Quantitative PCR analysis after Crip2 knockdown (**C**) and overexpression (**D**). Knockdown reduced Slc31a1 and increased Slc25a3, whereas reduced Slc25a3 after overexpression was observed in the Crip2-OE#2 condition. Data are mean ± SD from three independent biological experiments (*n* = 3; one-way ANOVA with Dunnett’s post hoc test). These assays quantify transcript abundance, and do not directly measure copper flux or cuproptosis. In (**A**), red and blue indicate positive and negative correlations, respectively; color intensity reflects correlation magnitude, and |r| is the absolute Spearman correlation coefficient. In (**C**,**D**), blue, tan, and gray identify the control and two perturbation groups, and points denote independent biological experiments. Abbreviations: GTEx, Genotype-Tissue Expression; qPCR, quantitative PCR; GAPDH, glyceraldehyde-3-phosphate dehydrogenase.

Across GTEx cardiovascular tissues, CRIP2 expression correlated with several copper-handling genes (Figure 4A). In the focused left-ventricle panel, 13 of 14 correlations remained significant after Benjamini–Hochberg adjustment; ceruloplasmin was the exception (Spearman r = 0.039; adjusted *p* = 0.580). Western blotting confirmed CRIP2 knockdown with two siRNAs and overexpression with two plasmids in H9c2 cells (Figure 4B, Figures S3 and S4). *Crip2* knockdown was accompanied by lower *Slc31a1* and higher *Slc25a3*, while *Cox17*, *Atp7a*, and *Atp7b* changed little or not significantly (Figure 4C). After overexpression, *Slc25a3* was lower with CRIP2-OE#2, but not consistently across both constructs (Figure 4D). These transcript-level associations do not establish intracellular copper changes, mitochondrial copper delivery, cuproptosis, or direct transcriptional regulation.

### 2.5. CRIP2 Overexpression Is Associated with Higher Metabolic Activity During Hypoxia/Reoxygenation

**Figure 5:**
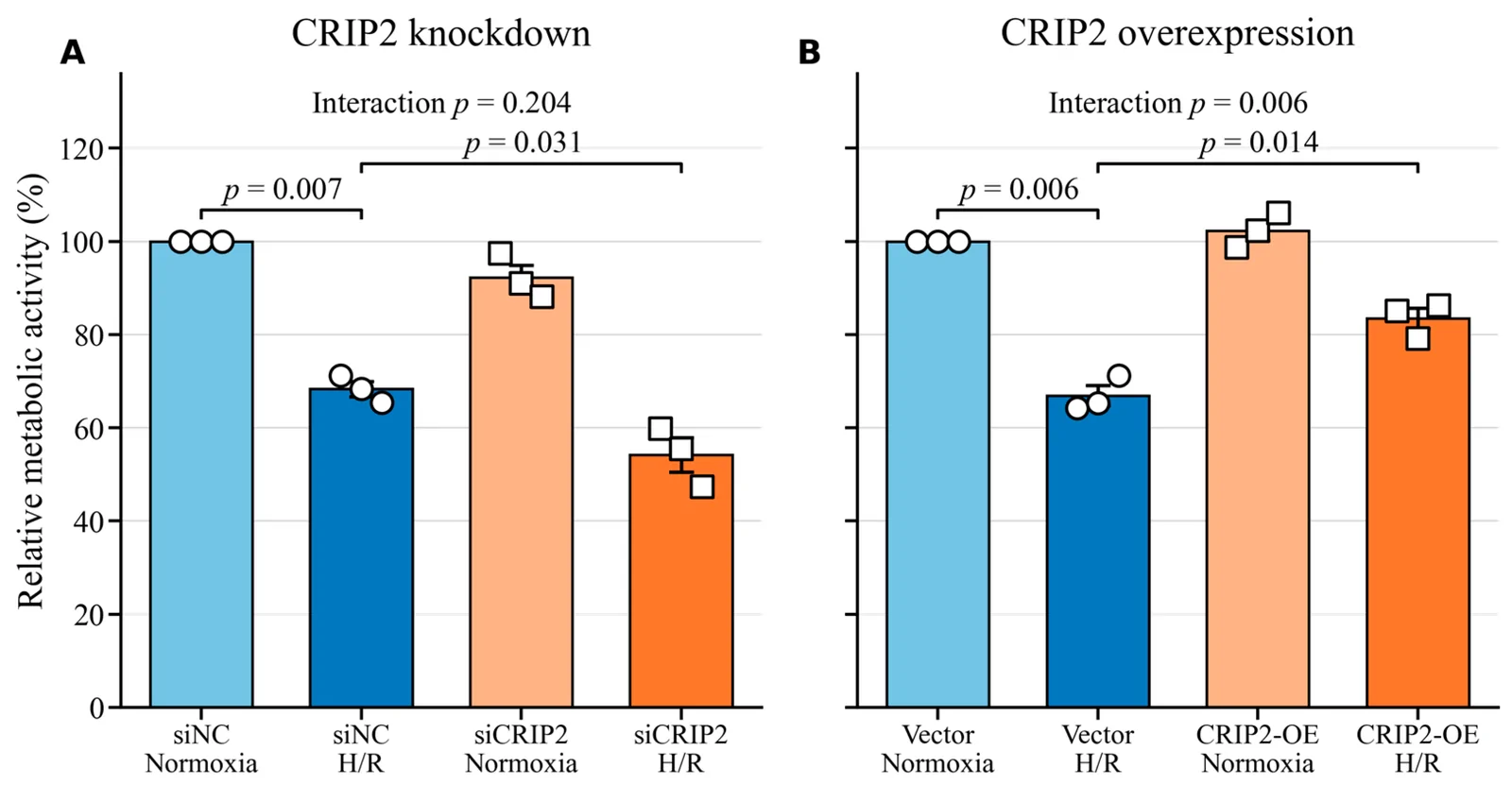
CRIP2 perturbation is associated with relative metabolic activity during hypoxia/reoxygenation in H9c2 cardiomyoblasts. (**A**) Knockdown module: siNC and siCRIP2 under normoxia or hypoxia/reoxygenation (H/R). (**B**) Overexpression module: empty vector and CRIP2-OE under normoxia or H/R. Bars show mean ± SEM, and open symbols show three independent biological experiments (*n* = 3); three technical wells were averaged within each experiment. Values were normalized within each experiment to the corresponding module-specific normoxic control. Displayed pairwise *p* values are from two-sided paired *t*-tests on blank-corrected mean OD450 values. Interaction *p* values are from blocked two-factor linear models containing CRIP2 treatment, oxygen condition, their interaction, and experimental batch as a block; knockdown and overexpression modules were analyzed separately. CCK-8 measures tetrazolium-reducing metabolic activity/relative viability, and does not directly measure cell death or cuproptosis. Blue and orange denote the module-specific control and CRIP2 perturbation, respectively; light and dark shades denote normoxia and H/R. CCK-8, Cell Counting Kit-8.

All 24 condition-by-experiment technical-replicate groups had coefficients of variation of 10% or less, and all raw wells were retained. H/R lowered relative metabolic activity in both control groups (siNC: 68.3 ± 1.7% versus 100.0%, *p* = 0.0070; vector: 66.9 ± 2.2% versus 100.0%, *p* = 0.0061; Figure 5). In the knockdown module, siCRIP2 values were 92.2 ± 2.7% under normoxia (*p* = 0.115 versus siNC) and 54.2 ± 3.7% under H/R (*p* = 0.031 versus siNC under H/R). Treatment and oxygen main effects were significant, but their interaction was not (*p* = 0.204); the data therefore do not show a knockdown-specific increase in H/R susceptibility. In the overexpression module, CRIP2-OE values were 102.3 ± 2.1% under normoxia (*p* = 0.380 versus vector) and 83.5 ± 2.2% under H/R (*p* = 0.014 versus vector under H/R). The treatment-by-oxygen interaction was significant (*p* = 0.00551), indicating an overexpression-associated difference in CCK-8 metabolic activity during H/R.

### 2.6. H/R qPCR Identifies an Atp7a Interaction That Remains Significant After Correction

**Figure 6:**
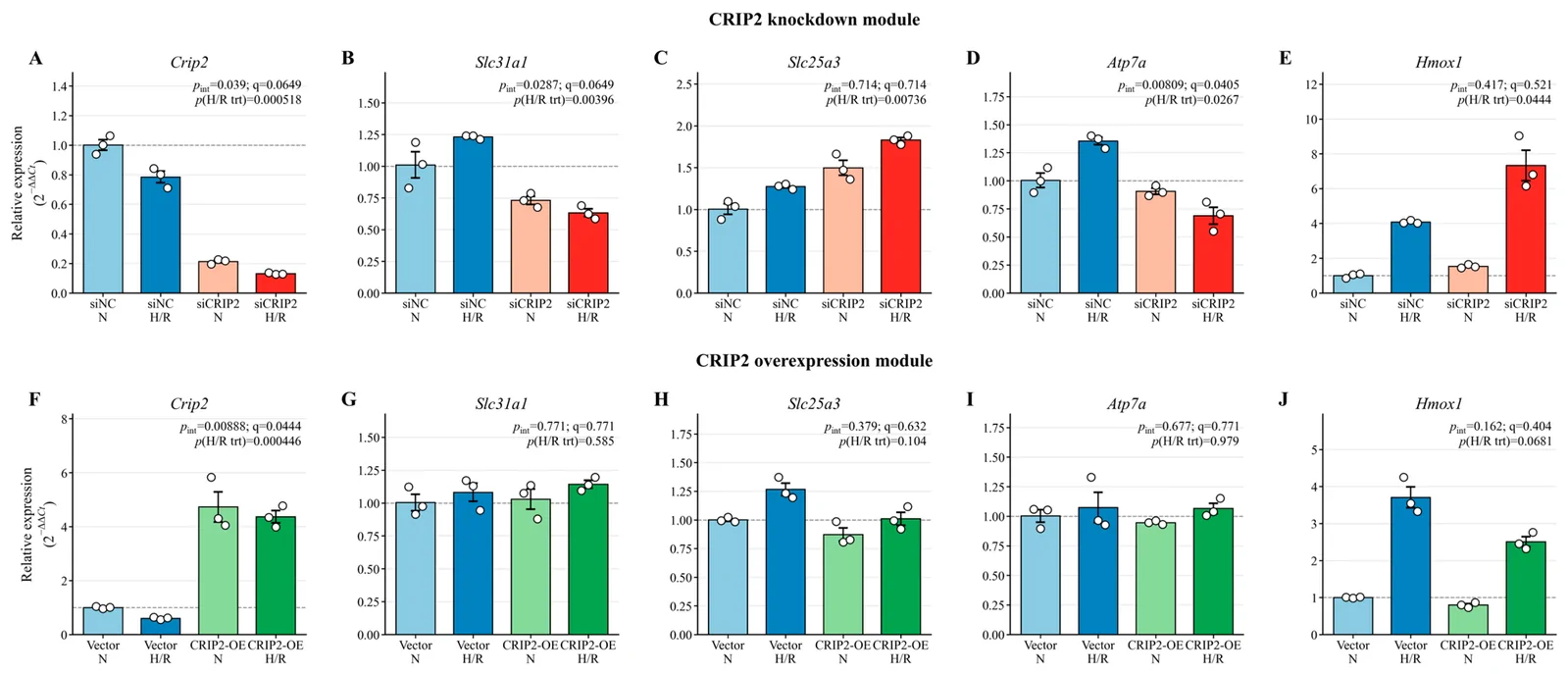
CRIP2 perturbation is associated with context-dependent copper-handling and oxidative-stress transcriptional responses during hypoxia/reoxygenation in H9c2 cardiomyoblasts. (**A**–**E**) Knockdown module: siNC and siCRIP2 under normoxia (N) or hypoxia/reoxygenation (H/R). (**F**–**J**) Overexpression module: empty vector and CRIP2-OE under normoxia or H/R. Crip2, Slc31a1, Slc25a3, Atp7a, and Hmox1 were quantified relative to Gapdh using the 2^−ΔΔCt^ method. Bars show mean ± SEM, and open symbols show three independent biological experiments (*n* = 3); three technical wells were averaged within each experiment. Interaction *p* values are from blocked two-factor linear models on ΔCt values containing CRIP2 treatment, oxygen condition, their interaction, and biological batch as a block. Interaction q values are Benjamini–Hochberg-adjusted across five genes within each module. *p*(H/R treatment) denotes the descriptive two-sided paired comparison between CRIP2 perturbation and the corresponding control under H/R, and is not multiplicity-adjusted. Blue bars denote module-specific controls, red bars denote siCRIP2, and green bars denote CRIP2-OE; light and dark shades denote normoxia and H/R, respectively. Open circles show independent biological experiments.

All 144 sample-by-gene technical triplicates had Ct standard deviations of 0.5 or less; no well was excluded. *Gapdh* Ct values showed no significant treatment, oxygen, or interaction effect. *Crip2* expression confirmed knockdown (0.214-fold under normoxia; 0.131-fold under H/R) and overexpression (4.730-fold under normoxia; 4.368-fold under H/R; Figure 6). Under H/R, siCRIP2 cells had lower *Slc31a1* (0.633-fold versus 1.231-fold in siNC; paired *p* = 0.00396) and *Atp7a* (0.689-fold versus 1.355-fold; *p* = 0.0267), and higher *Slc25a3* (1.830-fold versus 1.275-fold; *p* = 0.00736) and *Hmox1* (7.330-fold versus 4.078-fold; *p* = 0.0444). Only the *Atp7a* treatment-by-oxygen interaction survived Benjamini–Hochberg correction across the five targets (q = 0.0405); the *Crip2* and *Slc31a1* interactions were nominal, and the *Slc25a3* and *Hmox1* interactions were not significant. In the overexpression module, *Slc31a1*, *Slc25a3*, and *Atp7a* were not significantly different between CRIP2-OE and vector under H/R. *Hmox1* was lower with CRIP2-OE (2.511-fold versus 3.709-fold), but the paired comparison (*p* = 0.0681) and interaction (q = 0.404) were not significant. The response was therefore gene- and context-specific, not a uniform normalization of the panel.

## 3. Discussion

The most reproducible observation was lower myocardial *Crip2* after MI: it appeared in the local LAD-ligation cohort and in an independent bulk-tissue time course. The latter dataset also showed negative enrichment of a prespecified lipoylated-tricarboxylic-acid module. The broader copper-homeostasis set was not enriched, however, and no copper-dependent death endpoint was measured. The enrichment result therefore describes a transcriptional state compatible with reduced lipoylated-TCA activity, not cuproptosis itself. The MR estimate for CRIP2 was directionally consistent across sensitivity analyses and was based on strong instruments, but it remained nominal, relied mainly on trans-pQTLs, and was not supported by the single available cis-window candidate. We use it only to help prioritize CRIP2, not to infer causality.

CRIP2 had a reasonable biological basis for follow-up. Proteomic and biochemical work identified it as a nuclear copper-binding partner of ATOX1 and linked copper-dependent CRIP2 turnover to reactive oxygen species and autophagy [20]. *Crip2* deletion in skeletal muscle cells was subsequently associated with copper accumulation and impaired differentiation [26]. Separately, vascular studies implicated CRIP2 in endothelial aggregation, migration, proliferation, and angiogenesis [19]. This literature connects CRIP2 to copper handling and cardiovascular cell biology, but it does not show that the same mechanism operates in infarcted myocardium.

Circulating copper has been studied as a systemic cardiovascular measure [18], and ceruloplasmin has also been evaluated in cardiovascular cohorts [27]. Our analyses addressed a different question: regional myocardial expression and cardiac cell-type localization. Blood measurements cannot identify the myocardial compartment from which a signal arises, while tissue data do not establish a useful circulating biomarker. Post-MI remodeling is spatially organized across inflammatory, fibrotic, vascular, and metabolic programs [28]. Because myocardial CRIP2, circulating CRIP2, copper, and ceruloplasmin were not measured in the same subjects, their relationship and comparative diagnostic value remain unknown.

The perturbation experiments provide a narrower signal than a copper-regulation claim. *Slc31a1* encodes a high-affinity copper importer [29], and *Slc25a3* contributes to mitochondrial copper delivery for cytochrome c oxidase assembly [30]. CRIP2 has reported transcriptional effects [31], and *Crip2* deficiency alters cellular copper handling in skeletal muscle cells [26], so changes in these transcripts are biologically plausible. Even so, transcript abundance cannot reveal the direction or magnitude of copper flux. A model in which CRIP2 shifts copper uptake or mitochondrial allocation remains testable, but would require direct measurements of labile and mitochondrial copper, respiration, lipoylated-protein aggregation, iron–sulfur protein loss, and copper-dependent death [32]. Until those data exist, CRIP2 is a candidate for mechanistic study, rather than a validated copper regulator or therapeutic target [33].

The H/R qPCR data reinforce this restricted interpretation. *Atp7a* was the only copper-handling transcript with an interaction that remained significant after correction in the knockdown module. Lower *Slc31a1* and higher *Slc25a3* and *Hmox1* under H/R were supported by descriptive pairwise comparisons, but their interactions did not survive correction. Overexpression did not significantly normalize *Slc31a1*, *Slc25a3*, or *Atp7a*, and the lower *Hmox1* value under H/R was a non-significant trend. The pattern is consistent with context-dependent transcriptional responses, not coordinated pathway rescue.

CCK-8 added a limited functional phenotype. H/R reduced tetrazolium-reducing activity in both control modules. Knockdown was associated with lower activity, but the non-significant interaction does not support selective sensitization to H/R. Overexpression was associated with higher activity during H/R and a significant interaction. With three independent experiments, this is preliminary evidence of an overexpression-linked metabolic phenotype. It cannot distinguish cell number from metabolic state, identify a death pathway, or connect the phenotype to intracellular copper.

Current cardiovascular literature places cuproptosis within wider mitochondrial quality-control and metabolic-stress networks [34,35]. Our gene-set result fits that context, but the study did not measure lipoylated-protein aggregation, iron–sulfur protein loss, respiration, or copper-dependent cell death [32]. We therefore treat the proposed CRIP2–copper–cuproptosis link as a hypothesis, not as an observed pathway.

Any clinical implication remains speculative. The data do not support CRIP2 as a standalone biomarker or treatment target. A useful next step would be to determine whether myocardial or circulating CRIP2 adds information beyond infarct size, ventricular function, copper, and ceruloplasmin, and whether blood CRIP2 reflects myocardial expression. Such questions require prospective cohorts with paired blood and tissue measurements before CRIP2-directed stratification or intervention can be considered.

The evidence is constrained at several levels. Public and local datasets differ in platform, species, and processing. The MR screen used a suggestive threshold and mainly trans-pQTLs; no protein passed Bonferroni correction, the single cis-window candidate was null, and the available exposure data did not permit Steiger testing or colocalization. The local animal comparison included five mice per group, limiting precision. Two nominal MI single-cell matrices were byte-identical, leaving three sham and two unique MI hearts for pseudobulk sensitivity analysis; the atlas therefore supports localization, not replicated condition-level inference. Geneformer and scTenifoldKnk were exploratory, were not significantly concordant, and no topology effect remained significant after adjustment. H9c2 cardiomyoblasts also have limited resemblance to adult cardiomyocytes [36].

## 4. Materials and Methods

### 4.1. Myocardial Datasets

We analyzed expression profiles from the infarcted myocardium (IM), peri-infarct zone (PZ), and non-infarcted myocardium (NI) using the mouse MI dataset GSE775 [37]. Sample inclusion followed the original annotations. The accession details and sample characteristics are summarized in Tables S1 and S2. Differentially expressed genes (DEGs) from these regional MI contrasts were intersected with DEGs identified in our in-house mouse MI RNA-seq cohort, generated after LAD coronary artery ligation.

For independent bulk-tissue validation, we reanalyzed GSE236374, comprising three sham, three MI day-7, and three MI day-28 adult male C57BL/6JR hearts [25]. Raw gene counts were converted to log2 counts per million for sample-level visualization. The archived DESeq2 MI day-7-versus-sham Wald statistics were used for gene-level inference. A prespecified seven-gene cuproptosis-permissive/lipoylated-tricarboxylic-acid core module (Fdx1, Lias, Lipt1, Dld, Dlat, Pdha1, and Pdhb), a 14-gene copper-homeostasis set, and three negative regulators were tested by preranked gene set enrichment analysis with 10,000 phenotype-independent gene-set permutations and a fixed random seed. Gene-wise z scores were averaged only for descriptive sample-level module plots; formal inference was based on DESeq2 and preranked enrichment statistics.

Tissue expression matrices were obtained from the Genotype-Tissue Expression (GTEx) project. Pairwise Spearman correlations between CRIP2 and curated copper-handling genes were computed across the left ventricle, atrial appendage, coronary artery, and aorta, and visualized as bubble heatmaps. For the focused left-ventricle 14-gene panel, *p* values were additionally adjusted by the Benjamini–Hochberg procedure. Mouse cardiac single-cell RNA-sequencing data (GSE214611; GEO metadata nominally listed three sham and three MI accessions) were processed using Seurat. Cells with fewer than 200 or more than 5000 detected genes, or with mitochondrial transcripts exceeding 15%, were excluded, yielding 96,288 cells (34,959 sham and 61,329 MI). Counts were normalized and log-transformed, highly variable genes were selected, and reciprocal principal-component analysis was used for integration. Principal-component analysis and Uniform Manifold Approximation and Projection used dimensions 1–30, with clustering resolution 0.5. Clusters were manually annotated using canonical markers for cardiomyocytes, fibroblasts, endothelial cells, macrophages, and smooth muscle cells/pericytes [38]. Before biological-replicate inference, the downloaded matrix, feature, and barcode files were compared by SHA-256 checksum. GSM6613074_snd7_1 and GSM6613075_snd7_2 were byte-identical for all three file types; consequently, unique-heart pseudobulk sensitivity analysis used three sham and two unique MI matrices, with exact two-sided permutation *p* values. Cell-level atlas summaries were retained only as descriptive localization evidence.

### 4.2. In Silico Perturbation and Network Vulnerability Analyses

Geneformer was used to model virtual CRIP2 knockout and overexpression in MI and sham single-cell contexts. For each CRIP2 perturbation, downstream shifts among curated copper-handling and mitochondrial respiratory genes were summarized by embedding cosine distance. Orthogonal topology analysis with scTenifoldKnk estimated CRIP2-knockout-derived network vulnerability Z-scores in the same contexts. Across 13 genes, the Spearman association between the Geneformer knockout embedding shift in MI and the scTenifoldKnk MI topology Z-score was used as an exploratory cross-method comparison; a two-sided *p* value and a percentile 95% confidence interval from 100,000 bootstrap resamples were reported. This analysis was not prespecified as evidence of mechanistic concordance.

### 4.3. Local LAD Coronary Ligation Model and Tissue Collection

Male mice were subjected to LAD coronary artery ligation under isoflurane anesthesia, to induce MI. The sham controls underwent the same surgical exposure without coronary ligation. Hearts were harvested four weeks after surgery, rinsed in cold buffer, and dissected for RNA extraction and qPCR validation. All animal experiments were conducted in accordance with institutional guidelines and approved by the Institutional Animal Care and Use Committee of Guangzhou Medical University (approval no. N2025-02004).

### 4.4. RNA Extraction, Library Preparation, and Bulk RNA-Seq

Total RNA was isolated, treated with DNase, and assessed for its integrity. Poly(A) libraries were sequenced on an Illumina platform (Illumina, Inc., San Diego, CA, USA) using paired-end reads. Adapters and low-quality bases were trimmed, reads were aligned to the appropriate reference genome using STAR, and gene-level counts were obtained using feature counts. Normalization was performed using the median-of-ratios method, and low-abundance genes were filtered prior to statistical testing (Table S3).

### 4.5. Differential Expression and Cross-Model Integration

DEGs were computed for IM versus NI and PZ versus NI in the regional MI dataset, and for LAD/MI versus sham in the in-house mouse RNA-seq cohort, using DESeq2 with Benjamini–Hochberg adjustment. To derive tissue-anchored candidates, MI regional DEGs were intersected with LAD/MI DEGs, retaining genes with consistent ischemic injury responsiveness across datasets. Functional enrichment was performed using Gene Ontology and Kyoto Encyclopedia of Genes and Genomes libraries using clusterProfiler. Enrichment outputs were displayed as Venn diagrams, forest plots, dot plots, bar plots, and related figure panels, as appropriate.

### 4.6. Protein QTL Instruments and MI Outcome GWAS for Mendelian Randomization

pQTL summary statistics were obtained from the deCODE study, which profiled 35,559 Icelandic participants using SomaScan and reported associations for approximately 4907 circulating proteins. Instruments were constructed for proteins encoded by the intersecting differentially expressed genes when independent pQTLs were available at the suggestive threshold (*p* < 5 × 10^−6^) [39]. These instruments were not restricted to cis-pQTLs; for CRIP2, most selected variants were trans-pQTLs. The analysis is therefore interpreted as protein-level genetic prioritization, rather than direct cis-protein causal evidence. MI outcome data were obtained from the IEU Open GWAS resource (ebi-a-GCST011364), corresponding to Hartiala et al. and comprising 14,825 MI cases and 380,970 controls of European ancestry [40].

### 4.7. Two-Sample Mendelian Randomization

Two-sample Mendelian randomization (MR) used pQTLs as instruments for circulating protein levels and MI as the outcome. Instruments were selected at *p* < 5 × 10^−6^, clumped at r^2^ < 0.001 within a 10,000 kb window, and harmonized by effect allele. The inverse-variance weighted (IVW) estimator was primary, with weighted-median and MR-Egger estimates as sensitivity analyses. Instrument strength was assessed using F statistics. Heterogeneity was quantified using Cochran’s Q; directional pleiotropy was evaluated using the MR-Egger intercept; and leave-one-out and MR-PRESSO global/outlier analyses assessed influential variants and horizontal pleiotropy. Nominal *p* < 0.05 was used for candidate prioritization. Because approximately 32 proteins were screened, *p* < 0.00156 was additionally considered a Bonferroni sensitivity threshold; no candidate met this corrected threshold. For CRIP2, we also repeated IVW analysis at the conventional exposure threshold *p* < 5 × 10^−8^, and evaluated the only available ±1 Mb cis-window candidate by a Wald ratio. Formal Steiger directionality testing could not be estimated because exposure-effect allele frequencies and exposure sample size were unavailable; full cis-window summary statistics were also unavailable for colocalization. Analyses were implemented in R using TwoSampleMR and MR-PRESSO, and the instrument lists, harmonization logs, and MR outputs are provided in Table S4.

### 4.8. Cell Culture and Treatments

H9c2 cardiomyoblasts were maintained in high-glucose Dulbecco’s modified Eagle medium (DMEM) supplemented with 10% fetal bovine serum and antibiotics at 37 degrees C in 5% CO_2_. Cells were transfected under matched culture conditions for CRIP2 knockdown or overexpression experiments, with non-targeting small interfering RNA (siRNA) or empty vector controls run in parallel.

### 4.9. CRIP2 Perturbations

For loss of function, two independent non-overlapping small interfering RNAs (siRNAs) targeting Crip2 and a non-targeting control siRNA were transfected with a lipid-based reagent at standard working concentrations. For gain of function, two CRIP2 expression plasmids and empty-vector controls were transfected under matched conditions. Knockdown and overexpression efficiencies were verified by Western blotting and qPCR. Exact siRNA sequences and plasmid details are listed in Table S5.

### 4.10. Quantitative PCR

RNA was reverse-transcribed and quantified using SYBR Green qPCR. Glyceraldehyde-3-phosphate dehydrogenase (Gapdh) served as the internal reference. For in vivo validation, Crip2, Ccdc80, and Glipr2 were quantified in LAD and sham hearts. For the baseline H9c2 perturbation experiments, Crip2 and copper-handling genes, including Slc31a1, Slc25a3, Cox17, Atp7a, and Atp7b, were quantified. For the H/R experiments, Crip2, Slc31a1, Slc25a3, Atp7a, and Hmox1 were measured in the two parallel knockdown and overexpression modules. Each biological sample was measured in three technical wells; the technical Ct values were averaged before biological-level analysis. ΔCt was calculated as Ct(target) − Ct(Gapdh), and relative expression was calculated using the 2^−ΔΔCt^ method with the corresponding module-specific normoxic control mean as calibrator. Primer sequences and species are detailed in Table S6A,B.

### 4.11. Western Blotting

Cells were lysed in radioimmunoprecipitation assay (RIPA) buffer supplemented with protease and phosphatase inhibitors. Equal protein amounts were separated by sodium dodecyl sulfate–polyacrylamide gel electrophoresis (SDS-PAGE) and transferred to polyvinylidene difluoride (PVDF) membranes. Membranes were blocked and incubated with primary antibodies against CRIP2 and GAPDH, followed by horseradish peroxidase (HRP)-conjugated secondary antibodies and chemiluminescence detection. Representative blots were used to confirm the knockdown and overexpression efficiency in H9c2 cells. GAPDH served as the loading control.

### 4.12. Hypoxia/Reoxygenation and CCK-8 Assay

H9c2 cells were evaluated in two parallel 2 × 2 modules: siNC or siCRIP2 under normoxia or H/R, and empty vector or CRIP2 overexpression (CRIP2-OE) under normoxia or H/R. Cells were seeded at 5000 cells per well in 96-well plates (100 μL per well). Approximately 24 h after transfection, H/R plates were exposed to 1% O_2_ for 12 h followed by 6 h of reoxygenation under normoxia; paired control plates remained under normoxia for the same 18 h interval. Cell Counting Kit-8 reagent (Beyotime Biotechnology, Shanghai, China, catalog no. S1075S; 10 μL per well) was then added, and absorbance at 450 nm was measured after 2 h at 37 °C. Each condition contained three technical wells, and the complete experiment was performed in three independent biological replicates. Cell-free wells containing medium and CCK-8 served as plate-specific blanks. The mean blank absorbance was subtracted from each well, technical wells were averaged, and values were normalized within each experiment to the corresponding module-specific normoxic control. The CCK-8 readout was interpreted as relative tetrazolium-reducing metabolic activity/viability, rather than as a direct measure of cell death.

### 4.13. Statistical Analysis

Data are presented as mean ± standard error of the mean (SEM) for animal qPCR validation and as mean ± standard deviation (SD) for cell culture qPCR experiments, as indicated in the figure legends. Two-group comparisons were performed using two-tailed unpaired Student’s *t*-test. Multiple-group comparisons were performed using one-way analysis of variance (ANOVA) followed by Tukey’s or Dunnett’s post hoc test, as appropriate. Exact *p* values are shown in the figures, and statistical significance was set at *p* < 0.05. For CCK-8 analysis, the knockdown and overexpression modules were evaluated separately. Blank-corrected technical wells were averaged to yield one value per condition in each independent experiment. A blocked two-factor linear model included CRIP2 treatment, oxygen condition, their interaction, and an experimental batch as a block. Prespecified within-batch comparisons were assessed using two-sided paired *t*-tests on blank-corrected mean OD450 values and treated as descriptive, without multiplicity adjustment. Relative values were used for visualization, and technical wells were not treated as independent biological replicates. For the H/R qPCR analysis, knockdown and overexpression modules were analyzed separately. Inferential tests were performed on ΔCt values using a blocked two-factor linear model containing CRIP2 treatment, oxygen condition, their interaction, and a biological batch as a block. Interaction *p* values were adjusted by the Benjamini–Hochberg procedure across the five target genes within each module. Prespecified within-batch comparisons were assessed using two-sided paired *t*-tests on ΔCt values and are reported descriptively, without multiplicity adjustment. Relative 2^−ΔΔCt^ values were used for visualization, and technical wells were not treated as independent replicates.

## 5. Conclusions

CRIP2 emerged as a reproducible tissue-derived candidate associated with myocardial copper-handling responses after MI. H/R experiments identified one adjusted-significant *Atp7a* interaction after knockdown and an overexpression-associated CCK-8 phenotype, but did not establish altered copper flux, cuproptosis, or myocardial remodeling. Direct copper and mitochondrial measurements, orthogonal cell-death assays, adult or human cardiomyocyte models, and myocardium-specific in vivo perturbation are needed to test the proposed mechanism.

## Data Availability

The data supporting the findings of this study are available from the corresponding author upon reasonable request.

## Supplementary Materials

The following supporting information can be downloaded at: https://www.mdpi.com/article/10.3390/cimb48080767/s1.

## Abbreviations

The following abbreviations are used in this manuscript:

ATOX1	Antioxidant 1 Copper Chaperone
CM	Cardiomyocytes
CRIP2/Crip2	Cysteine-rich intestinal protein 2
CCDC80	Coiled-coil domain containing 80
CCK-8	Cell Counting Kit-8
COX17	Cytochrome c oxidase copper chaperone COX17
DALYs	Disability-adjusted life years
DEG(s)	Differentially expressed gene(s)
EC	Endothelial cells
FB	Fibroblasts
GAPDH	Glyceraldehyde-3-phosphate dehydrogenase
GLIPR2	GLI pathogenesis-related 2
GTEx	Genotype-Tissue Expression
H/R	Hypoxia/reoxygenation
GWAS	Genome-wide association study
H9c2	Rat embryonic cardiomyoblast cell line
IHD	Ischemic heart disease
IM	Infarcted myocardium
KO	Knockout
LAD	Left anterior descending (coronary artery)
Mac	Macrophages
MI	Myocardial infarction
MR	Mendelian randomization
NI	Non-infarcted myocardium
OE	Overexpression
pQTL	Protein quantitative trait locus
PZ	Peri-infarct zone
qPCR	Quantitative polymerase chain reaction
ROS	Reactive oxygen species
scRNA-seq	Single-cell RNA sequencing
SLC25A3/Slc25a3	Solute carrier family 25 member 3
SLC31A1/Slc31a1	Solute carrier family 31 member 1
SMC/Pericyte	Smooth muscle cells/Pericytes
SOD1	Superoxide dismutase 1
UMAP	Uniform Manifold Approximation and Projection
ANOVA	Analysis of variance
CI	Confidence interval
DMEM	Dulbecco’s modified Eagle medium
HRP	Horseradish peroxidase
IVW	Inverse-variance weighted
MR-PRESSO	Mendelian Randomization Pleiotropy RESidual Sum and Outlier
PVDF	Polyvinylidene difluoride
SD	Standard deviation
SDS-PAGE	Sodium dodecyl sulfate–polyacrylamide gel electrophoresis
SEM	Standard error of the mean
siRNA	Small interfering RNA
BH	Benjamini–Hochberg
GSEA	Gene set enrichment analysis
TCA	Tricarboxylic acid
