# Supplementary material for: Integrative Analysis Prioritizes CRIP2 as a Candidate Associated with Myocardial Copper-Handling Responses After Myocardial Infarction
Source: Curr Issues Mol Biol. 2026 Jul 28;48(8):767. doi: 10.3390/cimb48080767 (PMC13511074; doi:10.3390/cimb48080767)
Supplement: Supplementary file 1 [file cimb-48-00767-s001.zip › cimb-4424136 Supplementary Figures.pdf]

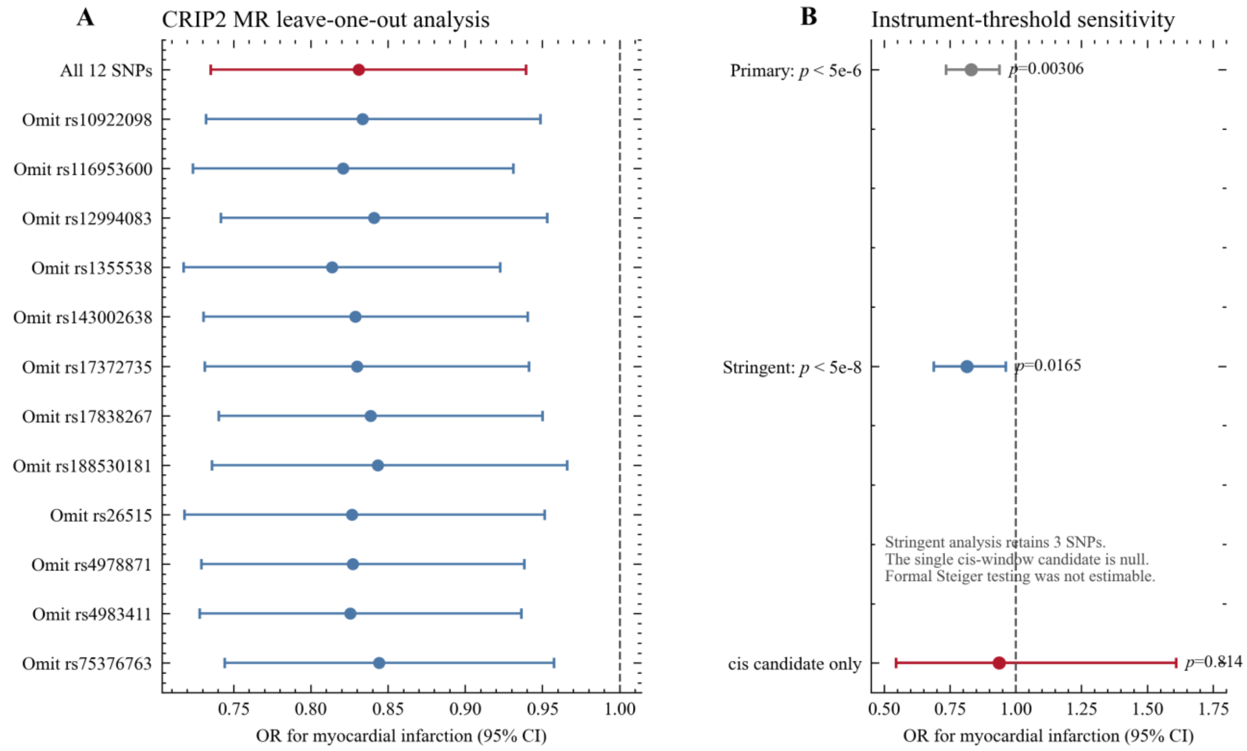

Figure S1. CRIP2 Mendelian randomization sensitivity analyses. (A) Leave-one-out inverse-variance-weighted estimates remained directionally consistent after each instrument was omitted (OR range = 0.8138–0.8443; maximum p = 0.0140). (B) The primary 12-SNP model yielded OR = 0.8310 (95% CI 0.7352–0.9393; p = 0.00306; heterogeneity p = 0.8998). The stringent three-SNP model yielded OR = 0.8147 (95% CI 0.6891–0.9633; p = 0.0165; heterogeneity p = 0.9170). The available ±1-Mb cis-window candidate, rs4983411, was null (Wald-ratio OR = 0.9369; 95% CI 0.5453–1.6098; p = 0.8135). Steiger testing and colocalization were not estimable from the available exposure summary data. The MR result was used for candidate prioritization, not as definitive causal evidence.

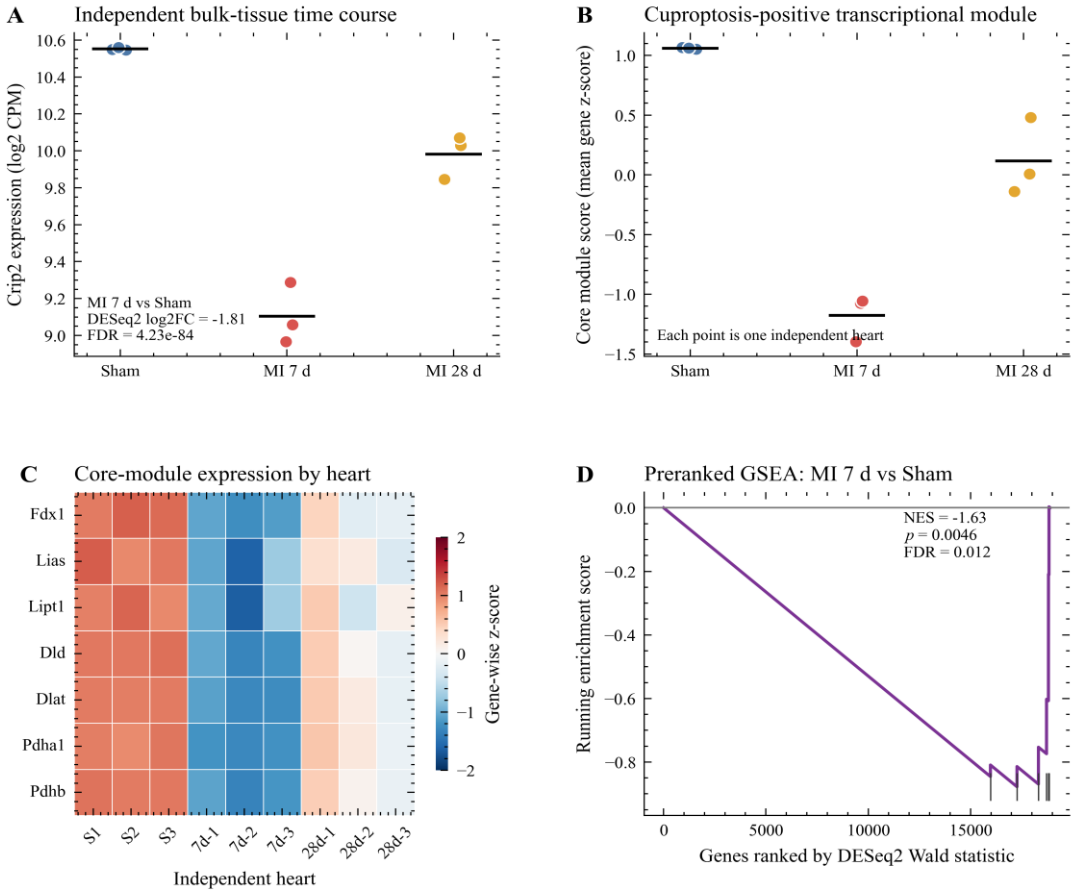

Figure S2. Independent bulk-heart time-course validation and preranked gene-set enrichment analysis. (A) Crip2 expression across Sham, MI day 7, and MI day 28 hearts in GSE236374; each point represents one independent heart (n = 3 per time point). DESeq2 showed lower Crip2 expression at MI day 7 versus Sham (log2 fold change = −1.8104; adjusted p = 4.23 × 10−84). (B) Sample-level score for the prespecified lipoylated-tricarboxylic-acid core module. (C) Gene-wise standardized expression of Fdx1, Lias, Lipt1, Dld, Dlat, Pdha1, and Pdhb. (D) Preranked gene-set enrichment analysis for MI day 7 versus Sham showed negative enrichment of the core module (NES = −1.6325; nominal p = 0.00457; FDR = 0.0121). The broader 14-gene copper-homeostasis set was not significant (NES = −1.2945; FDR = 0.2361). These data describe transcriptional suppression and do not demonstrate cuproptotic cell death.

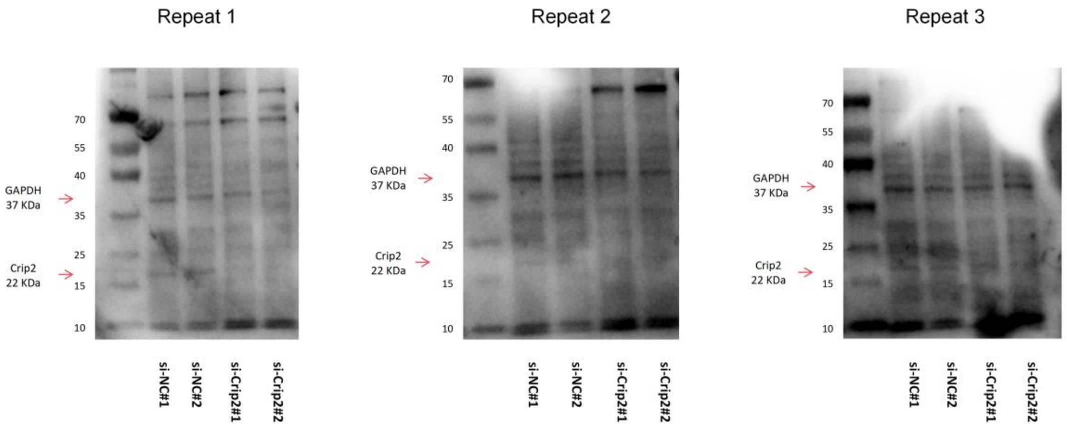

Figure S3. Original western blot images of CRIP2 knockdown in H9c2 cells across three independent experiments. Full, uncropped western blot images from three independent biological experiments (Repeat 1–3) are shown. CRIP2 (~22 kDa) was assessed in H9c2 cells transfected with negative-control siRNAs (si-NC#1 and si-NC#2) or Crip2-targeting siRNAs (si-Crip2#1 and si-Crip2#2). GAPDH (~37 kDa) served as the loading control. Primary antibodies were anti-CRIP2 (Proteintech Group, Inc., Rosemont, IL, USA; catalog no. 14801-1-AP) and anti-GAPDH (Proteintech Group, Inc., Rosemont, IL, USA; catalog no. 60004-1-Ig).

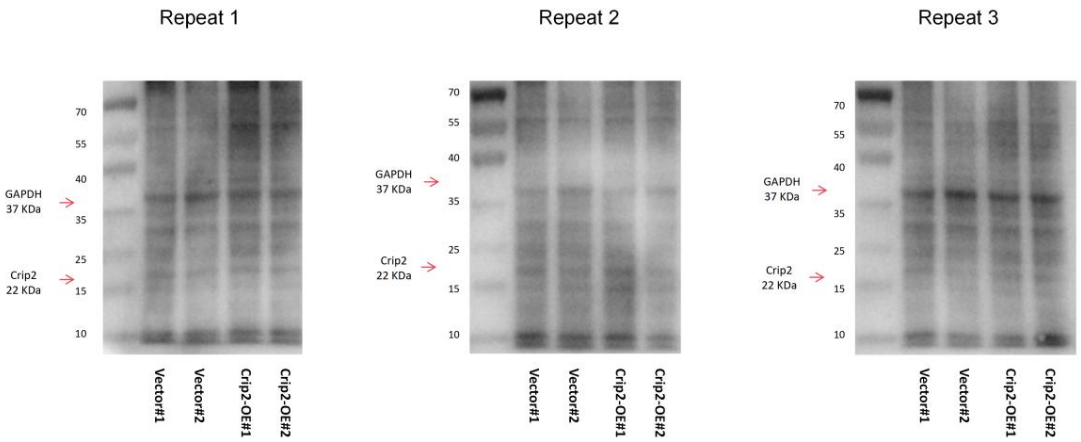

Figure S4. Original western blot images of CRIP2 overexpression in H9c2 cells across three independent experiments. Full, uncropped western blot images from three independent biological experiments (Repeat 1–3) are shown. CRIP2 (~22 kDa) was assessed after transfection with empty control vectors (Vector#1 and Vector#2) or Crip2 overexpression plasmids (Crip2-OE#1 and Crip2-OE#2). GAPDH (~37 kDa) served as the loading control. Primary antibodies were anti-CRIP2 (Proteintech Group, Inc., Rosemont, IL, USA; catalog no. 14801-1-AP) and anti-GAPDH (Proteintech Group, Inc., Rosemont, IL, USA; catalog no. 60004-1-Ig).
